# Transarterial embolization (TAE) of sacral giant cell Tumor (GCT) using spherical parmanent embolic material superabsorbant polymer microsphere (SAP-MS)

**DOI:** 10.1186/2193-1801-2-666

**Published:** 2013-12-11

**Authors:** Katsuyuki Nakanishi, Keigo Osuga, Shinichi Hori, Kenichiro Hamada, Nobuyuki Hashimoto, Nobuhito Araki, Hideki Yoshikawa, Noriyuki Tomiyama

**Affiliations:** Department of Diagnostic Radiology, Osaka Medical Center for Cancer and Cardiovascular Diseases, 1-3-3, Nakamichi, Higashinari-ku Osaka, 537-8511 Japan; Department of Diagnostic and Interventional Radiology Osaka University Graduate School of Medicine, Osaka, Japan; Gate Tower Institute for Image Guided Therapy, Osaka, Japan; Department of Orthopedic Surgery, Osaka University Graduate School of Medicine, Osaka, Japan; Department of Orthopedic Surgery, Osaka Medical Center for Cancer and Cardiovascular Diseases, Osaka, Japan

**Keywords:** Giant cell tumor, Sacrum, Embolization, Microspheres

## Abstract

**Purpose:**

We retrospectively evaluated our experience of transcatheter arterial embolization (TAE) of the sacral GCT with use of a spherical permanent embolic agent, superabsorbant polymer microsphere (SAP-MS) as an alternative treatment modality.

**Materials and methods:**

From 1997 to 2011, four patients with sacral GCT were treated with TAE. In all cases, SAP-MS was used as an embolic material. The effects of TAE were evaluated for improvement of patients’ symptoms, radiographic change such as vascularity of tumor, size of tumor and occurrence of reossification.

**Results:**

Of the four patients, three responded favorably to TAE with improvement in pain and neurologic symptoms with long-term follow up. Diminished vascularity, stabilization of tumor size and reossification were shown radiographically. One patient died because of tumor growth 26 months after the initial TAE.

**Conclusion:**

In sacral GCT, TAE using SAP-MS might be useful for symptom improvement, reossification of the lesion and stabilization of tumor size.

## Introduction

Giant cell tumor (GCT) of bone is an aggressive benign neoplasm (Lackman et al. 2002) which occurs most frequently at the end of long bones. The sacrum is the fourth most common site, accounting for between 1.7–8.2% of cases (Martin and McCarthy. 2010). With GCT of long bones, curretage and/or surgical exision is usually a first-line treatment. However, with sacral GCT, the size and location of the tumor make surgical treatment difficult and dangerous.

Therefore, various non-surgical treatment methods have been advocated including transarterial embolization(TAE), radiation, and cryotherapy (Martin and McCarthy 2010). There have been many reports about arterial embolization of sacral GCT (Lackman et al. 2002; Martin and McCarthy 2010; Lin et al. 2002; Hosalkar et al. 2007; Onishi et al. 2010; Thangaraj et al. 2010).

In these literatures, TAE has mainly been used either as a method of preoperative devascularization or as a palliative measure for inoperable lesions. Recently, the development of permanent spherical embolic materials such as Hepasphere (Merit Medical, Rockland, MA, USA), Embosphere (Merit Medical, Rockland, MA, USA), BeadBlock (Biocompatibles, UK) and so on (Abramowitz et al. 2009) have been remarkable. These materials have been widely used for embolization of hepatocellular carcinoma, uterine fibroid and arteriovenous malformation (Abramowitz et al. 2009; Maeda et al. 2012; Osuga et al. 2008; Khankan et al. 2004; Laurent et al. 2004) and so on. However, to the best of our knowledge, there have been only a few reports of use of spherical embolic agents for musculoskeletal tumors (Amendola et al. 2013; Basile et al. 2004). We employed spherical permanent embolic material superabsorbant polymer microsphere (SAP-MS) for palliative TAE in four cases of sacral GCT for diminishing vascularity, promoting reossification and as a result, to accomplish pain relief and improvement of activities of daily life (ADL).

We retrospectively evaluated the effect of TAE of sacral GCT with SAP-MS.

## Materials and methods

### Patients

Between 1997 and 2011, four patients (2 males; 2 females) with sacral GCT underwent TAE 15 times totally (Table 1). In all patients, the diagnosis of GCT was confirmed histologically by previous biopsies or operation. One patient had already undergone radiotherapy about three months before initial TAE (case 1); one patient had a recurrent tumor and had undergone curretage about six months before initial TAE (case 4) in a previous treatment; one patient had undergone radiotherapy about three months after final TAE as an additional treatment (case 2). Before performing TAE, all patients complained of skeletal pain which had impaired their ADL and two patients needed morphine sulfate. In all patients, International Society of Limb Salvage (ISOLS) criteria to the lower extremity was less than 50% (Tables 1 and 2) (Enneking et al. 1993).

**Table 1:** **Patients’ list and clinical outcome**

Case	Age	Gender	Previous Tr.	Additional Tr.	Session of TAEs	Follow-up(M)	ISOLS score		Radiographic change					Vs
									Tumor size(cm)		Volume change (%)	Reossification	Embolized arteries	
							Before TAE	After TAE	Before TAE	After TAE				
1	30	M	RT 50 Gy 3 M before initial TAE		3	118	17%	93%	9 × 9 × 7	5 × 5 × 4	-82.4	(+)	Bil. LSAs, Lt-5th LA	Alive
2	68	F		RT 50 Gy 3 M after final TAE	3	141	17%	80%	9 × 9 × 8	6 × 6 × 4	-77.8	(+)	Bil. LSAs	Alive
3	40	F			4	39	33%	96%	12 × 10 × 10	9 × 7 × 9	-52.3	(+)	Bil. LSAs	Alive
4	32	M	Curretage 6 M before initial TAE		5	14	46%	died	7 × 7 × 9	9 × 12 × 18	340.8	(-)	Bil. LSAs	DOD

Tr. = treatment, RT = radiation therapy, M = months.

average (case1-3) = 99.3, M = months.

average (case1-3) = -70.8.

LSA = Lateral sacral artery, LA = Lumbar artery, Bi. = bilateral.

Vs = Vital status.

**Table 2:** **ISOLS criteria specific to the lower extremity (Enneking et al.** 1993 **)**

Score	Pain	Function	Emotional acceptance	Support	Walking ability	Gait
**5**	No pain	No restriction	Rnthused	None	Unlimited	Normal
**4**	Intermediate	Intermediate	Intermediate	Intermediate	Intermediate	Intermediate
**3**	Modest/Non-disabling	Recreational restriction	Satisfied	Brace	LImited	Minor cosmetic
**2**	Intermediate	Intermediate	Intermediate	Intermediate	Intermediate	Intermediate
**1**	Moderate/intermittently disabling	Partial occupational restriction	Partial occupational restriction	One cane or crutch	Inside only	Major cosmetic
**0**	Severe/continuosly disabling	Total occupational restriction	Total occupational restriction	Two cane or crutch	Not independently	Major handicap

The mean tumor size was more than 9 cm diameter. In all cases, the tumors were evaluated as hypervascular as contrast enhanced dynamic MRI. All cases were judged as inoperable and TAE was considered to be an exclusive treatment modality in a palliative setting.

### Embolic material properties

SAP-MS is characterized by a spherical shape of uniform size, deformability, biocompatibility, non-biodegradability and ease in delivering through microcatheter.

It has a unique property of an ability to absorb fluids within minutes and increase its diameter. The diameter in an ionic contrast material (Ioxaglate 320 mg/ml) and human serum is approximately 2 to 3.5 times larger than its original size. The particle size, calibrated every 50 μm, ranged from 53 to 350 μm. Animal study showed limited reactive and inflammatory changes in the arterial wall and surrounding tissue without later recanalization (Abramowitz et al. 2009; Maeda et al. 2012; Osuga et al. 2008; Khankan et al. 2004; Laurent et al. 2004). SAP-MS was originally invented by one of the co-authors (Jiaqi et al. 1996), and was approved in June 2013 as Hepasphere in our country, but it was not commercially available at the time we treated these four patients. Therefore, the prototype material was used during the study period, and the use of the material in embolization procedures was permitted and approved by our institutional committee on a patient-by-patient basis. All patients gave informed consent to undergo the embolization procedure. The same material had been already approved as HepaSphere in Europe and QuadraSphere in USA.

This retrospective study was also approved by our institutional review board (IRB).

### Methods

Baseline digital subtraction angiography (DSA) was performed by a 4.2-F diagnostic catheter for identifying the feeding arteries. After that, a 2.3-F microcatheter was advanced coaxially into the peripheral portion of each feeding artery as close to the tumor as possible. Selective DSA was performed to confirm the feeding arteries were supplying the target lesion. The embolized arteries included the lateral sacral, iliolumbar, fifth lumbar and medial sacral arteries (Table 1).

Neither systemic analgestic sedation nor prophylatic antibiotics were used.

1–2 ml of 1% lidocaine was infused before embolization to prevent the embolic local pain.

SAP-MS particles suspended in sodium meglumine ioxaglate 320 mgl/ml with concentration of 10 mg/ml were injected through a microcatheter slowly under fluoroscopy guidance. Smaller particles (53–106, 106–150 μm) were chosen at first, followed by larger particles (150–212 μm).

The endpoint of embolization with SAP-MS was the disappearance of the majority of tumor stain due to intratumoral vessel occlusion. Gelatin sponge (GS) particles were secondarily used to plug the proximal portion of the feeding artery until complete cessation of the blood flow could be seen under fluoroscopy guidance. As a rule, no chemotherapeutic agent was used.

Additional sessions of embolization were performed when skeletal pain recurred or radiological tumor regrowth was recognized.

### Evaluation and monitoring the patients’ symptom before and after TAE

The changes in symptoms after TAE were evaluated by retrospective review of medical charts using the ISOLS evaluation form and the written record of the symptoms (Table 2) by three orthopedic surgery co-authors. It is based on the analysis of following the six factors (pain, functional activities, emotional acceptance, external supports, walking abilities and gait) (Enneking et al. 1993). For each of the six factors, values of 0 to 5 are assigned based on established criteria. For each factor, specific values (0,1,3,or 5) are equated with certain levels of achievement or performance. Intermediate values of 2 or 4 are assigned, based on the examiner’s judgment, when achievement or performance falls between the specified values. When each of the factors has been scored, the sum of the individual factor scores is recorded.

The maximum possible score (number of factors multiplied by five is recorded and the rating percentage is determined by dividing the maximum score into the total score (Enneking et al. 1993). The radiographic change after TAE was evaluated for occurrence of reossification by X-ray image or CT and size reduction calculated three dimensionally by MR images.

The ISOLS score and imaging outcomes were assessed in a final examination.

## Results

The patients’ backgrounds and clinical outcomes are summarized in Table 1.

**Figure 1:**
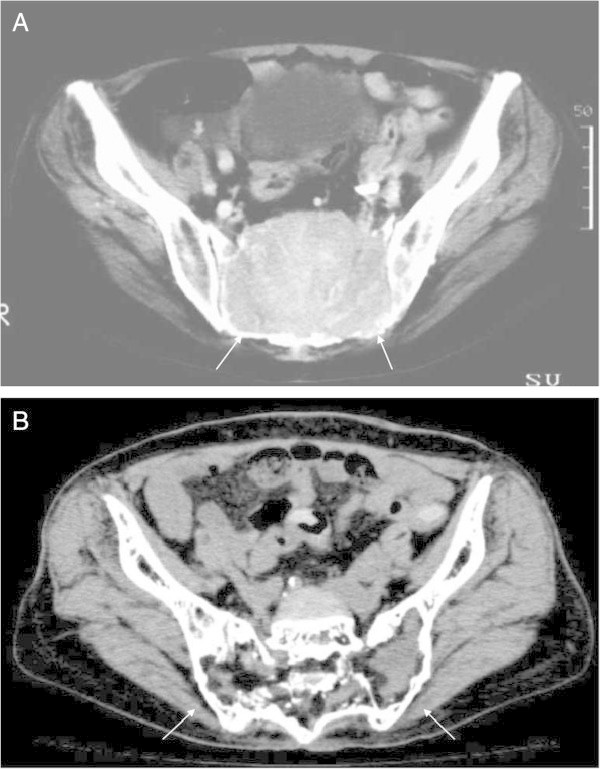
**A 68-year-old female with GCT in the sacrum (Case 2). A.** On CT, a large osteolytic mass occupied the sacrum (white arrows). **B.** On CT of same level as Figure 1A about 10 years after initial TAE, the size of the mass decreased and obvious reossification is shown (white arrows).

**Figure 2:**
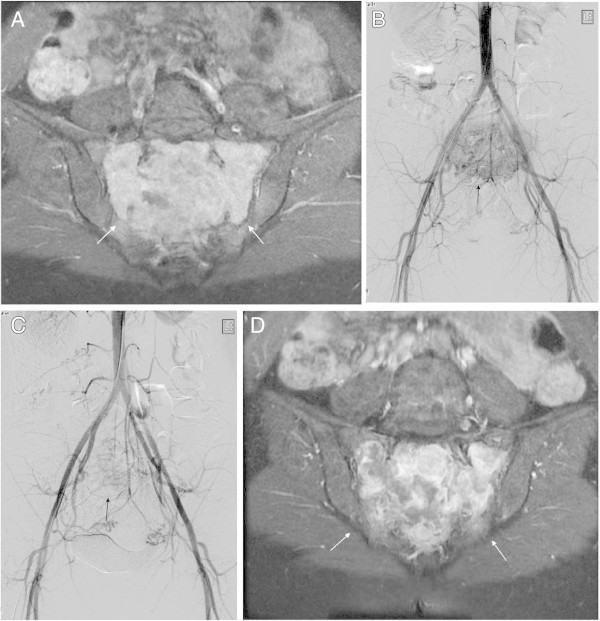
**A 40-year-old female with GCT in the sacrum (Case 3). A.** On contrast enhanced fat supressed T1-weighted image of MRI, the homogenous enhanced mass occupied the sacrum (white arrows). **B.** On digital sabtraction pelvic angiogram, the hypervascular mass is shown in the sacrum (arrow). **C.** On digital sabtraction pelvic angiogram after TAE of bilateral lateral sacral arteries and middle sacral artery, the tumor stain is diminished (arrow). **D.** On contrast enhanced fat supressed T1-weighted image of MRI about 14 months after initial TAE, the enhanced pattern becomes inhomogenous compared with Figure 2A, which shows what appears to be a necrotic effect (white arrows).

All procedures (total 15 TAEs, mean three times) were technically successful. The interval between each TAE ranged from one month to nine months. In three patients, the ISOLS scores after the last TAE session were more than 80%, and their symptoms remained improved throughout the follow-up periods. In these three patients, mean volume reduction was 71% (range, 52.8%–82.4%), and obvious reossification was observed after the final TAE (Figures 1 and 2).

One patient died 14 months after the initial treatment because of tumor growth in spite of repeated TAEs (case 4). In this case, pain relief and improvement of ADL were only transient after TAE.

Post embolization syndromes (fever, local pain, or nausea) remained in a conservatively controllable range.

In one case (Case 1), permanent left-peroneal nerve pulsy was observed after the third TAE, where the left L5 lumbar artery was embolized.

## Discussion

Sacral GCT often presents as massive tumors (Lin et al. 2002). The size and location of the tumor make surgical treatment difficult and dangerous. Radiation could be an alternative treatment in the case of surgically unfavorable sizes and locations (Lin et al. 2002), but secondary sarcoma, although rare, can arise after radiation therapy in such a wide radiation field (Nakanishi et al. 2001). Therefore, TAE can be considered as one of the treatment options (Lackman et al. 2002; Martin and McCarthy 2010; Lin et al. 2002; Hosalkar et al. 2007; Onishi et al. 2010; Thangaraj et al. 2010). There are several reports on TAE of sacral GCT. However, only a few reports focus on the embolic materials or their comparison (Amendola et al. 2013; Basile et al. 2004).

Early reports (Lackman et al. 2002; Lin et al. 2002; Hosalkar et al. 2007; Onishi et al. 2010) described that gelfoam, polyvinyl alcohol (PVA) particles and stainless steel coil for TAE of sacral GCT were used. These conventional materials were also used for pelvic tumors including metastasis from renal cell carcinoma and aneurysmal bone cyst (Chuang et al. 1979). However, these embolic materials have the following problems.

GS particles have the potential of short-term (weeks to a month) proximal occlusion of a tumor-feeding artery (Maeda et al. 2012; Osuga et al. 2008; Khankan et al. 2004). PVA is the most commonly used permanent embolic agent. However, the irregular-shaped PVA particles tend to clump together leading to proximal vessel occlusion, which is potentially the risk of non-target embolization and future collateral development to the target lesion. In addition, the organized thrombus in the spaces among PVA particles can be partially recanalized. Basile et al. (Basile et al. 2004) compared the efficacy of trisacryl gelatine microsphere (TGM) versus PVA in the preoperative embolization of bone neoplasms, and intraoperative blood loss volume was significantly lower after embolizatioon with TGM than after that with PVA. SAP-MS was originally designed for TAE of hypervascular tumors. Osuga et al. described its inertness and efficacy in TAE of large hepatocellular carcinoma (HCC) (Osuga et al. 2008; Laurent et al. 2004). Precisely calibrated SAP-MS particles can penetrate intratumoral vessels leading to long-term tumor devascularization, whereas, the blood flow into the surrounding tissues can be spared.

Such a targeted TAE using SAP-MS minimizes the risk of ischemic damage of the normal tissues as shown by histological evaluation.

Similarly, the use of calibrated microspheres have been used to treat hypervascular tumors such as HCC as well as uterine fibroid (Abramowitz et al. 2009; Maeda et al. 2012; Osuga et al. 2008; Khankan et al. 2004; Laurent et al. 2004). Our primary aim was to accomplish more peripheral arterial occlusion at the level of intra- or peri-tumoral vessels using spherical embolic agents. GS particles were secondarily used to plug the proximal feeding artery until complete cessation of the blood flow was obtained. In our study, metalic coils were not used to allow selective catheterization in the subsequent embolization. Instead, the long term duration of devascularization and symptom improvement were obtained by a relatively small number of TAE sessions. In the previous reports, TAE was repeated on schedule every 4–6 weeks until no new vessel formation was evident (Lackman et al. 2002; Hosalkar et al. 2007). In contrast, in our cases, excluding the one fatal case, only three or four sessions of TAE were needed during the follow-up period ranging 39–118 months (mean 99.3 mo). Favorable long-term outcomes exceeding 100 months were obtained in two cases (Case 1 and 2), although radiation therapy was added prior to or after TAE. In one case (Case 1), left-peroneal nerve pulsy was observed after embolization of left L5 lumbar artery, probably because of the ischemic damage of the corresponding tissue.

In one case (case 4), pain relief and ADL improvement were only transient after TAE. TAE could not control the tumor progression, and the patient died 14 months after the initial TAE. In this case, the tumor stain consisted of fine vessels, and disappeared immediately after only a small amount of embolic material was injected. As a result, the necrotic effect was minimal on contrast-enhanced MRI, and the tumor size increased rapidly. We speculate that feeder occlusion at the proximal level allowed collateral circulation to the residual tumors.

There are several limitations in this study. First, this is a retrospecitve study with a small number of cases. In two cases, the effectiveness of TAE may be influenced by combination of the radiation therapy. There are no established scores to evaluate the clinical conditions related to the sacral GCT.

In conclusion, TAE can be considered as the treatment option of choice for sacral GCT either alone or in conjunction with other therapy. In our experience, SAP-MS, a spherical embolic agent, was a safe and effective material to achieve peripheral tumor vessel occlusion resulting in long-term symptom improvement and tumor size reduction.
